# Comparative Evaluation of Biomass Power Generation Systems in China Using Hybrid Life Cycle Inventory Analysis

**DOI:** 10.1155/2014/735431

**Published:** 2014-10-14

**Authors:** Huacai Liu, Xiuli Yin, Chuangzhi Wu

**Affiliations:** CAS Key Laboratory of Renewable Energy, Guangzhou Institute of Energy Conversion, Chinese Academy of Sciences, Guangzhou 510640, China

## Abstract

There has been a rapid growth in using agricultural residues as an energy source to generate electricity in China. Biomass power generation (BPG) systems may vary significantly in technology, scale, and feedstock and consequently in their performances. A comparative evaluation of five typical BPG systems has been conducted in this study through a hybrid life cycle inventory (LCI) approach. Results show that requirements of fossil energy savings, and greenhouse gas (GHG) emission reductions, as well as emission reductions of SO_2_ and NO*_x_*, can be best met by the BPG systems. The cofiring systems were found to behave better than the biomass-only fired system and the biomass gasification systems in terms of energy savings and GHG emission reductions. Comparing with results of conventional process-base LCI, an important aspect to note is the significant contribution of infrastructure, equipment, and maintenance of the plant, which require the input of various types of materials, fuels, services, and the consequent GHG emissions. The results demonstrate characteristics and differences of BPG systems and help identify critical opportunities for biomass power development in China.

## 1. Introduction

With the rapid development of economy, electricity demand continues to grow in China. Significant attention has been focused on the high dependence of electricity generation on coal, including greenhouse gases (GHG) emissions and environmental pollution. Alternate approaches are being sought. Biomass is the only renewable fuel available for combustion-based electricity generation. Moreover, straw is no longer the primary fuel for cooking and space heating in many rural communities. A large amount of straw is abandoned or incinerated in the fields, resulting in environmental pollution and a waste of resources. For these reasons, biomass power generation (BPG) has gained significant attention in China. By the end of 2010, the overall installed capacity of biomass power had reached 5.5 GW. In addition, China has set the goals in State Plans for Medium and Long-Term Development of Renewable Energy to achieve 30 GW of biomass power capacity by 2020 [1].

There are currently three kinds of biomass power plants in China: biomass-only fired power plant, biomass-coal co-firing power plant, and biomass gasification power plant. These plants may vary significantly in technology, scale, and feedstock and consequently in their performances. In the short term, large-scale electricity generation based on biomass-only fired or cofiring is the most promising alternative to achieve expected biomass electricity market contribution and to fulfill GHG emissions targets, mainly due to better technological reliability and maturity [2]. However, biomass is usually characterized by massive volume and dispersed distribution, which may incur high energy and economic costs during collection. Small- and medium-scale biomass gasification power generation technology may be more feasible than large-scale direct-fired and cofiring technology [3, 4]. A full assessment and comparison of potential of each system for sustainable development should be conducted.

Several studies have been undertaken using life cycle assessment (LCA) to analyze benefits and drawbacks of BPG systems in China because LCA considers all the processes involved in each alternative in a cradle-to-grave manner [5–8]. However, direct comparisons on the same bases are difficult to find. Therefore, five typical power plants are studied in this study through a detailed life cycle inventory (LCI) approach: 25 MW biomass-only fired plant, 140 MW cofiring plant, 25 MW cofiring plant, 1 MW gasification plant, and 5.5 MW gasification plant. The results demonstrate characteristics and differences of BPG systems and help identify critical opportunities for biomass power development in China.

## 2. Methodology

LCI analysis is one of the four phases of LCA involving the compilation and quantification of inputs and outputs. The two basic methods for compiling an LCI are the process-based analysis and the input-output (IO) analysis. Most LCIs have been conducted based on a process-based analysis where the physical quantities of energy and material use and environmental releases from the main production processes are assessed in detail, but nevertheless the process-based analysis suffers from a systematic truncation error due to the delineation of product system by a finite boundary and the omission of contributions outside the boundary [9]. In contrast, input-output analysis is a top-down technique that uses sectoral monetary transaction matrixes describing complex interdependencies of industries within a national economy. Input-output analysis can overcome the “truncation error” and solve the traditional system boundary limitation by taking into account capital goods and overheads as inputs to a product system, which are often deliberately left out by most of process-based LCIs [9]. However, it also has limitations associated with high levels of aggregation in industry or commodity classifications, as well as potential uncertainty [9, 10]. Moreover, monetary value, the most commonly used representation of interindustry transactions in input-output tables, may distort physical flow relations between industries due to price inhomogeneity [9].

The hybrid LCI method seeks to use advantages of both methods while mitigating their respective limitations. One of the practical examples has been presented by Inaba and his coworkers [11]. He developed a production equilibrium hybrid model to assess reductions in CO_2_ emissions by food waste biogasification by using a matrix representing the input-output relationship of energy and materials among the processes and sectors. The method presented and utilized in this research takes a similar approach to that used by Inaba et al. [11]. To gain more insight into contributions of energy consumptions and emissions of BPG systems, each lifecycle stage was considered as a subsystem of the larger group of Chinese industrial sectors in the LCI model.

### 2.1. Goals and Scope

In a consequential LCA, the differences in environmental impact stemming from changes made to a reference system are quantified. Fossil fuel system substituted or most likely to be substituted by biomass energy system is usually chosen as the reference system. Compared with the reference system, biomass power plants' performances may vary depending on other factors such as the normal routes of biomass disposal and local energy consumption structure. For the sake of comparison, some simplifications have been made in this study. Credits are not taken for the avoided operations of normal routes of biomass disposal such as field burning. Coal-fired plant before retrofitting for cofiring is chosen to be the reference system of biomass cofiring systems. It was assumed that the specific portion of coal cofired with biomass has the same electric efficiency and emissions as those before cofiring. The differences of electric output and emissions between cofiring and the specific portion of coal cofired with biomass were allocated to biomass. Biomass is assumed to substitute part of the coal without changing the performance of the rest part of coal in power generation. A simple and useful comparison can be carried out this way. For the biomass-only system and biomass gasification system, the reference system is the electricity sector in IO table (see Table 3), which represents the national average of electricity production. Despite this, useful information can be obtained since power generation from coal accounts for more than 80% of annual power generation in China [12].

Two main objectives were pursued in this study: firstly, to determine the reduction of GHG emissions, the primary energy (PE) savings that could actually be attained when biomass power is compared to conventional electricity production, and secondly, to evaluate which BPG system is more beneficial on the same basis. We have established that the LCI is done from the production process of the biomass to the electric output suitable for consumption of power plants.

### 2.2. System Boundaries

**Figure 1:**
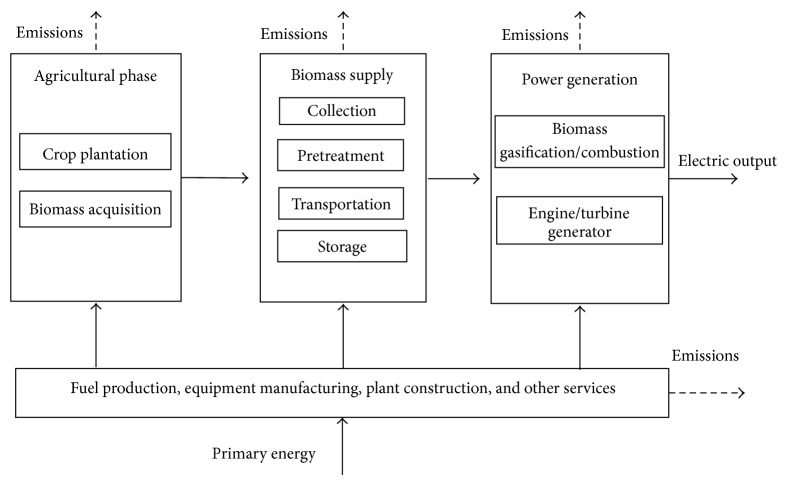
System boundaries of BPG system.

A consistent scope of system boundaries which mainly includes three life cycle stages is adopted to facilitate comparison between technologies, as shown in Figure 1. In all of the stages, the energy, material, and other services during the entire life time of the plant were considered, which includes supports from background economy such as fuel production, extraction/production of essential materials, and manufacturing and commissioning of equipment. The ash will be either disposed or used as industrial raw material and it is not taken into account in this study, neither are the end-use of electricity and the decommissioning of power plant.

### 2.3. Model Description

The previously described inventory data were summarized into an input-output format, and the energy and material flow between processes in the system was calculated. A make-use input-output framework was used to illustrate the BPG systems, as shown in Table 1.

Energy and materials in the system are defined as a process commodity sector (*c* = 1,…, *l*), and a process itself is defined as a process activity sector (*p* = 1,…, *m*). Thereby, matrix **U** ^*CP*^ represents input to each process activity (*p*) of a process commodity (*c*), and matrix **V** ^*PC*^ represents the quantity of output of a process commodity (*c*) from a process activity (*p*).

Energy and material inputs from IO sector are described as matrixes **U** ^*EP*^ and **U** ^*NP*^, respectively, which comprises the IO sector and the process activity sector in the BPG systems. The matrix **U** ^*EP*^ represents input to each process activity (*p*) from *a* energy sector (*i* = 1, 2,…, *n*_*e*_), and matrix **U** ^*NP*^ represents input to each process activity (*p*) from a nonenergy sector (*j* = 1, 2,…, *n*).

Output of commodities from a process activity sector in the system (*p*) to IO energy sector (*i*) and nonenergy sector (*j*) is shown as matrixes **V** ^*PE*^ and **V** ^*PN*^. The matrixes **U** ^*EE*^, **U** ^*NE*^, **U** ^*EN*^, and **U** ^*NN*^ denote annual commodity transactions based on the energy and monetary unit between IO sectors.

A production equilibrium model for hybrid LCI analysis was established using a matrix representing the input-output relationship of materials and energy among the processes and sectors described above. The accounting framework is shown in Table 2.

The matrices **B** ^*CP*^ and **D** ^*PC*^ = **V** ^*PC*^ were calculated using (1) and (2), respectively. The squarely arranged input coefficient **A**_*ff*_ derived from (3) is composed of the commodity sector in the BPG systems. Vector **G** is the total output of the process sectors. Vector **Q** is the total amount of commodities. The symbol “$\hat{\;\;\;}$” indicates a diagonal matrix. Consider the following:

(1) $$\begin{array}{c} B^{CP}=U^{CP}{\hat{G}}^{-1}, \end{array}$$

(2) $$\begin{array}{c} D^{PC}=V^{PC}{\hat{Q}}^{-1}, \end{array}$$

(3) $$\begin{array}{c} A_{ff}=B^{CP}D^{PC}. \end{array}$$

The matrices **A**_*ef*_ and **A**_*nf*_ are inputs from energy sector and nonenergy sector of the BPG systems, respectively, as described below:

(4) $$\begin{array}{c} A_{ef}=\left(U^{EP}{\hat{G}}^{-1}\right)D^{PC}+\left(-{\left(V^{PE}\right)}^{T}{\hat{G}}^{-1}\right)D^{PC},\\ A_{nf}=\left(U^{NP}{\hat{G}}^{-1}\right)D^{PC}+-\left({\left(V^{PN}\right)}^{T}{\hat{G}}^{-1}\right)D^{PC}, \end{array}$$

where *T* represents the transpose of a matrix.

The matrixes **A**_*ee*_, **A**_*en*_, **A**_*ne*_, and **A**_*nn*_ describe the intersectoral requirements in the background economy:

(5) (AeeAenAneAnn)=(UEEUEEUNEUNN)x^−1,x=(xExN).

Electricity was assumed to be the only product of BPG systems. Therefore, the total energy requirement matrix **L** can be written as follows:

(6) $$\begin{array}{c} L={\left(\begin{array}{ccc} I-A_{ff} & -A_{fe} & -A_{fn}\\ -A_{ef} & I-A_{ee} & -A_{en}\\ -A_{nf} & -A_{ne} & I-A_{nn} \end{array}\right)}^{-1}. \end{array}$$

Direct and indirect emissions within a system **y**_1_ and outside of the system **y**_2_ were calculated by premultiplying **L** with direct emissions data and postmultiplying by demand for the good in question, as represented by the following equation:

(7) $$\begin{array}{c} \left(\begin{array}{cc} y_{1} & y_{2} \end{array}\right)=\left(\begin{array}{cc} d_{1} & d_{2} \end{array}\right){\left(\begin{array}{ccc} I-A_{ff} & -A_{fe} & -A_{fn}\\ -A_{ef} & I-A_{ee} & -A_{en}\\ -A_{nf} & -A_{ne} & I-A_{nn} \end{array}\right)}^{-1}\left(\begin{array}{c} u\\ 0 \end{array}\right), \end{array}$$

where **d**_1_ and **d**_2_ are the emissions per unit activity of sector in the system and of the emissions per unit activity of sector *t* in the IO table and **u** represents the functional unit vector of the system.

## 3. Data Sources and Assumptions

### 3.1. Classification of IO Sectors and Sectoral Energy Consumptions

China's 2007 monetary input-output table [13], the latest data of China national economy, was used to construct the hybrid LCI model. To make full use of data from energy statistics and to overcome the price inhomogeneity of energy, the energy-related sectors in IO table were divided into 18 specific energy production sectors. The 2008 China Energy Statistical Yearbook [14] was used to calculate sectoral energy intensity vector. Moreover, the IO table was aggregated into a 24-nonenergy-sector format to be consistent with the Chinese energy statistics based on the standards of classification of national economic industries [15]. The 18 energy sectors and 24 nonenergy sectors are shown in Table 3. The sectoral energy consumptions were transformed from physical units to energy units with data from the yearbook [14]. It should be noted that the energy statistics were accomplished based on industry sectors while the IO table was established based on commodity sectors. Thus the data of energy statistics was transformed into commodity-sector based data before integrating into the IO table.

The energy consumptions of the 5 energy-related sectors in the 2008 China Energy Statistical Yearbook [14] are categorized into 18 kinds of energy. Correspondingly, the 5 energy-related sectors in IO table were divided into 18 specific energy sectors to be consistent with the Chinese energy statistics, as shown in Tables 3 and 4. The energy consumptions of the 5 energy-related sectors were allocated to the 18 specific energy sectors, as shown in Table 4. For example, energy consumption of petroleum and natural gas extraction was allocated to 1-8 crude oil and 1-16 natural gas. In other words, it was allocated to petroleum extraction and natural gas extraction. Most of the energy allocation was done on energy basis while some exceptions are listed in Table 4.

Some of the energy is used as feedstock into different industrial processes. The 2008 China Energy Statistical Yearbook gives the total nonenergy use in the industrial sectors. It was assumed that all the nonenergy use is in the chemical sectors [16]. The “other petroleum products” in all sectors and coke use in smelting of metals are assumed to be completely nonenergy use [16].

### 3.2. Sectoral Air Emissions

GHG emissions particularly CO_2_, CH_4,_ and N_2_O expressed as CO_2_ equivalent (CO_2_-eq) have been assessed in this study. In addition, SO_2_ and NO_*x*_ emissions were taken into consideration when carrying out comparisons between options. The SO_2_ emissions of industries recorded in the China statistics yearbook [17] were used for creating the sectoral intensity matrix. The current Chinese statistic system does not provide any national or sectoral data on GHG and NO_*x*_ emissions. Thus, the GHG and NO_*x*_ emissions were generally calculated by multiplying the energy data by emission factors.

Chinese specific values for the carbon emission factor of each fuel and the fraction of carbon oxidized for each fuel in each sector from Peters et al. [16] were used to construct the CO_2_ emissions data. Meanwhile, IPCC (Intergovernmental Panel on Climate Change) default emission factors of CH_4_ and N_2_O of fuel combustion in each sector [18] were used to calculate the sectoral CH_4_ and N_2_O emissions data. The GHG emissions of nonenergy use from industrial processes were also taken into consideration, including CO_2_ emission from smelting and pressing of ferrous metals, CH_4_ emission from enteric fermentation, and N_2_O emission from cropland [19], as shown in Table 5.

Based on the country specific values of sectoral NO_*x*_ emission factors [16] and the industrial outputs [20], NO_*x*_ emissions from the main industrial processes can be estimated, as presented in Table 6.

### 3.3. Agricultural Phase

Biomass power plants' main features vary depending on several factors: amount of available resources and their properties, pretreatments required, and generation technology employed. For the sake of comparison, some representative average characteristics in biomass production and supply had to be selected in this case.

Corn stover is considered as feedstock for biomass power plants in this study. Life cycle data for the production, collection, and transportation of the feedstock include the energy and emissions associated with fertilizers, herbicides, and fuel to operate harvesting equipment. Data for the agricultural phase for corn originate mainly from national statistics [21], which represents the national average in 2007 (Table 7). Inputs were assigned to corn stover based on the purchased price of stover and grain, which is 120 yuan/t and 1500 yuan/t, respectively [21].

Energy allocation was rejected as grain has been considered an alimentary product and not a fuel. It has been considered that agricultural residues resources would not be collected without an energy demand and no economic value would be obtained. For this reason, partitioning on an economic basis using the share in revenues (grain and straw) was the method finally chosen.

### 3.4. Biomass Supply

The agricultural production is mainly carried out based on households in China, which results in a small average planted area and thus scattered straw resources. Thus the feedstock supply has become a bottleneck for large-scale use. There are mainly two patterns for biomass supply in China, which can be referred to as centralized pattern and distributed pattern. The centralized pattern involves a centralized storage site by the plant which can receive straw and ensure the plant's operation. Straw is mainly collected by farmers manually and then delivered to storage site by tractors. The distributed pattern involves a bunch of straw-receiving stations, which also serve as intermediate storage sites where biomass is baled and stacked [22]. The distributed pattern was employed by the 25 MW biomass-only fired plant and the 140 MW cofiring plant, while the centralized pattern was employed by the 25 MW cofiring plant, the 1 MW gasification plant, and the 5.5 MW gasification plant. The model established in our early study [22] was adopted to calculate the inputs of biomass supply system, including fuel, machinery, labor, and other services. Major parameters for biomass supply are listed in Table 8.

### 3.5. Biomass Power Stations

The combustion characteristics of biomass are well-understood and already wildly used in biomass applications worldwide. However, the development of biomass-only fired technologies starts fairly late in China. Advanced oversea technology and equipment have been employed in most cases. In recent years, some domestically developed boilers have met the basic operating requirements and have been put into operation but the performance still needs to be confirmed. A 25 MW biomass power plant in Anhui province was taken as an example in this study, which mainly consists of a 130 t/h high-temperature and high-pressure steam boiler with vibrating grate and a condensing steam turbine generator unit.

On the other hand, the R&D activity of BPG technology started since the 1960s, characterized by rice hull gasification and power generation system with sizes from 60 to 200 kW. A number of demonstration plants have been erected over the past few decades and some of them have been in operation for several thousands of hours. A demonstration project of 1 MW circulating fluidized bed biomass power plant which was established by the Guangzhou Institute of Energy Conversion, Chinese Academy of Sciences in Putian, Fujian Province, in 1998, was the first project of a MW-scale biomass power plant in China. Many improvements had been made in the 1 MW system as compared to the former 200 kW system. However, the overall efficiency of 1 MW system is still less than 20% [12], mainly due to the limited efficiency of internal gas engine generator. The 1 MW gasification and power generation system in this study consisted of an air-blown fluidized bed gasifier, a combined gas cleaner, five 200 kW gas engines, and a wastewater treatment system. For the first time a more efficient system that combines gas engine and steam turbine is employed in the 5.5 MW project [12], which is located in Xinghua, Jiangsu Province. But there are problems that remain to be solved, including lack of proper gas purification process and short continuous working time of the engine system. The 5.5 MW gasification power plant mainly comprises an atmospheric CFB gasifier, a gas-purifying system, 10 sets of 450 kW gas engines, a waste heat boiler, a 1.5 MW steam turbine, wastewater treatment and ash discharging systems, and so forth [12]. The data of biomass gasification plants in this study were mainly obtained from site-specific data and information.

Compared to biomass-only fired plants, cofiring offers two important advantages. Firstly, cofiring can take advantage of the higher efficiency of large-scale coal-fired power plants, even though boiler efficiency may decrease. Secondly, investment costs required to achieve bioelectricity production might be greatly reduced. However, there is still no subsidiary policy to support biomass cofiring in China. Only a few demonstration cases exist, like the Shiliquan Power Plant and several others. In addition, biomass price keeps increasing rapidly, leading to enormous fuel costs, and therefore the scheme has not been adopted in other power plants in China. The Shiliquan Power Plant is the first cofiring plant in China. Essential facilities were commissioned in a 140 MW generator set and crushed biomass is pneumatically conveyed into two cyclone burners in the boiler. The Shiliquan Power Plant and a 25 MW cofiring plant were taken as an example in this study. Data of cofiring plants was mainly collected from a report [23].

The major parameters of biomass power plants are shown in Table 9.

### 3.6. Data Classification for Biomass Power Generation (BPG) Systems

Inputs of BPG system from background economy consist of fuels, machinery, and other essential facilities. Corresponding IO sectors in the LCI model mainly include diesel, electricity, transport equipment, and ordinary machinery, equipment for special purpose, as indicated in Table 10. It is difficult to precisely classify the inputs into IO sectors, since the inputs involve almost all economic sectors and some data is highly aggregated. Thus data classification in this study follows rules below.

1. All inputs with clear category were classified according to the standards of classification of national economic industries [15]. For instance, boiler and steam turbine generator come from the sector “ordinary machinery, equipment for special purpose.”
2. Inputs without clear category were classified into the most related sectors based on evaluations of engineers.
3. Maintenance inputs were classified into sectors of corresponding equipment and services.
4. Purchase cost of equipment consists of prime cost and freight and miscellaneous charges. The freight and miscellaneous charges were classified into sector “transport, storage, postal, and telecommunications services.”

## 4. Results and Discussion

### 4.1. Primary Energy (PE) Consumption

**Figure 2:**
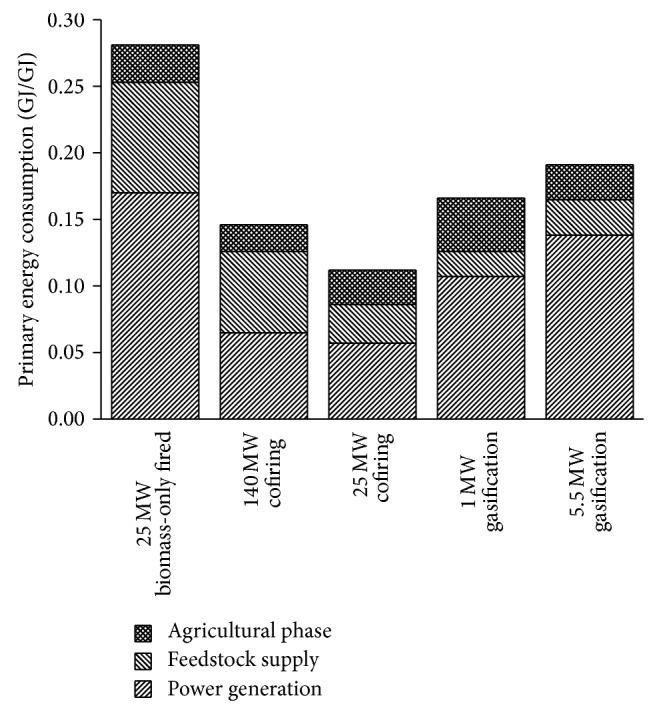
Comparison of PE consumptions of BPG systems.

PE consumption represents the sum of direct and indirect consumptions of fossil fuel energy associated with unit output of electricity from biomass. The PE consumption of BPG systems can also be defined as the fossil fuel energy consumed within the system per electric energy delivered to the utility grid. The results expressed in GJ/GJ are shown in Figure 2. All the values related to each life cycle process are included. Taking into account the inputs of agricultural phase, the PE consumption of BPG systems is 0.11–0.28 GJ/GJ. The PE consumption of the electricity sector obtained by the model is 2.85 GJ/GJ in this study. Thus, a large amount of PE can be saved by using biomass in power generation.

The 25 MW cofiring system exhibits a lower PE consumption, followed by the 140 MW cofiring system, the 1 MW gasification system, the 5.5 MW gasification system, and the 25 MW biomass-only fired in an ascending sequence: 0.11 < 0.15 < 0.17 < 0.19 < 0.28 GJ/GJ, respectively. Without the agricultural inputs, BPG systems appear to be of the same sequence of PE consumption, ranging from 0.09 GJ/GJ to 0.25 GJ/GJ. The major reason may be that cofiring systems avoid inputs of plant construction that are found in intensively invested system such as biomass-only fired system. Another reason is that depreciation of original coal power plant property has not been allocated to biomass power. The agricultural inputs play a noticeable role, especially in the 1 MW gasification system. PE consumption in Figure 2 of agricultural phase is 0.02–0.04 GJ/GJ, which becomes 0.01-0.02 GJ/GJ when the inputs of chemical fertilizers are removed. The chemical fertilizers account for over 50% of the PE consumption in agricultural phase.

The PE consumption of feedstock supply accounts for a significant portion in the case of the 25 MW biomass-only fired system and the 140 MW cofiring system (see Figure 2). Despite a higher electric efficiency, the 140 MW cofiring system consumes more PE than the 25 MW cofiring system, mainly owing to significant inputs in feedstock supply. The distributed pattern can ensure a quality and stable feedstock supply for large-scale BPG systems, but requires intensive investments for construction and operation of straw-receiving stations. In addition, fuel consumptions may also increase significantly as a result of pretreatments and additional intermediate handlings. On the other hand, the centralized pattern involves less investments and fuel consumption, which may be the first choice for small-scale systems. But more attention needs to be paid to road maintenance and utilization, for there would either be more capacity to be built or more traffic jams.

In addition to PE consumption, two other measures for assessing energy use can be defined:

(8) $$\begin{array}{c} \text{Energy}\;\text{savings}={\text{PE}}_{\text{ref}}-{\text{PE}}_{\text{bio}},\\ \text{Cost}\;\text{of}\;\text{energy}\;\text{savings}=\frac{C_{\text{bio}}}{{\text{PE}}_{\text{ref}}-{\text{PE}}_{\text{bio}}}, \end{array}$$

where PE_ref_ is the PE consumption of reference system, PE_bio_ is the PE consumption of BPG system, *C*_bio_ is the cost of biomass power generation, and *C*_bio_ can be expressed either in yuan/GJ or in GJ biomass/GJ. Due to the scarcity of biomass resources, the consumption of biomass may be regarded as another kind of cost.

The energy savings represent the amount of PE saved when unit electric energy from biomass is delivered to the utility grid. The cost of energy savings measures the amount of investment for every unit of PE saved by the BPG system. These two indicators may provide a better means of assessing the BPG systems. Comparing the results presented in Table 11, it can be observed that cofiring biomass resources in a coal power plant give better energy saving behavior than their conversion in biomass-only fired plant and biomass gasification plant. The highest energy savings are found in the 25 MW cofiring system, which means 4.42 GJ of PE can be saved when 1 GJ of electricity produced from biomass is delivered. The highest energy saving cost in yuan/GJ is found in the 25 MW biomass-only fired system, due to its intensive capital investments. On the other hand, the 1 MW gasification system appears to have a much higher energy saving cost in GJ biomass/GJ than the other BPG systems, as a result of low electric efficiency.

### 4.2. GHG Emissions

**Figure 3:**
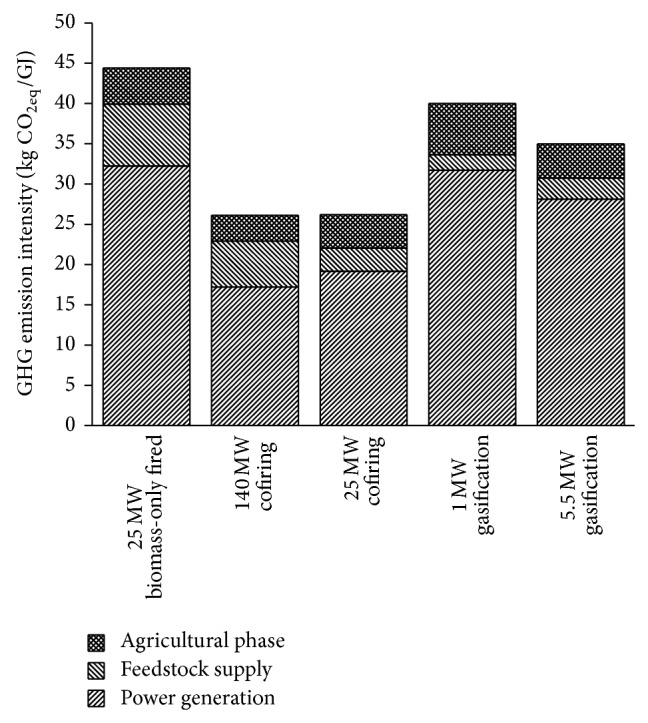
Comparison of GHG emission intensities of BPG systems.

The GHG emission intensity of BPG systems is defined as the GHG emission by the system per electric energy from biomass delivered to the utility grid, which includes the direct and indirect GHG emissions. By the commonly referred IPCC global warming potentials (CO_2_ :  CH_4_ :  N_2_O = 1 : 21 : 310), the GHG emissions of each BPG system assessed are shown in Figure 3. The GHG emission intensity of BPG systems is 26–44 kg CO_2_ eq/GJ, out of which the inputs of agricultural phase account for about 13%. A GHG emission intensity of 264 kg CO_2_ eq/GJ of the electricity sector is obtained in this study. Large amounts of GHG emission can be avoided by using biomass to substitute fossil energy in power generation. It should be noted that the 1 MW gasification system has a higher GHG emission intensity but a lower PE consumption than the 5.5 MW gasification system. Due to the lower electric efficiency, more biomass feedstock is consumed by the 1 MW gasification system per unit electric energy delivered, leading to more GHG emission from agricultural phase and biomass combustion.

For all systems, the majority of GHG emission comes from the life stage of power generation. N_2_O emission, specifically, from combustion of biomass fuels has significant contributions. In the cofiring cases, the results might differ widely from that obtained by the other researchers. According to Sebastián et al., biomass pretreatments account for more than 50% of biomass-related GHG emissions [2]. The major reason for this discrepancy may be that the energy consumption of feedstock crushing, which is the most energy-intensive operation, has been regarded as self-consumption of plants in this study. Other than that, the results are in good agreement with Liu et al. [8]. GHG emissions in the agricultural phase account for 10–16% of the total, mainly due to fertilizer production and the N_2_O emission caused by N-fertilizer utilization. Another important aspect that should be noticed is the significant contribution of infrastructure, equipment, and maintenance of the plant, which require the input of various types of materials, fossil fuels, and the consequent GHG emissions. For the 1 MW gasification system, the inputs of infrastructure, materials, and maintenance of the plant account for about 25% of the system's GHG emission. In some way, the importance of system completeness when conducting an LCA is testified. On the other hand, contributions of CO_2_ emissions from tractor operation during biomass transportation are relatively small.

Similar to the energy saving indicators, two other measures for assessing GHG emission reduction can be defined:

(9) $$\begin{array}{c} \text{GHG}\;\text{emission}\;\text{reductions}=\text{E}{\text{M}}_{\text{ref}}-\text{E}{\text{M}}_{\text{bio}},\\ \text{Cost}\;\text{of}\;\text{GHG}\;\text{emission}\;\text{reductions}=\frac{C_{\text{bio}}}{\text{E}{\text{M}}_{\text{ref}}-\text{E}{\text{M}}_{\text{bio}}}, \end{array}$$

where EM_ref_ is the GHG emission intensity of reference system and EM_bio_ is the GHG emission intensity of BPG system.

The GHG emission reductions represent the amount of GHG emission avoided per electric energy from biomass delivered to the utility grid. The cost of GHG emission reductions measures the amount of investment for every unit of GHG emission avoided by the BPG system. The cofiring plant performs better than the biomass-only fired plant and the biomass gasification plants in GHG emission reductions, as indicated in Table 12. The comparison results of GHG emission reductions are similar to that of energy savings, since the GHG emission reductions are roughly linear to the energy consumption savings.

### 4.3. SO_2_ and NO_*x*_ Emissions

**Figure 4:**
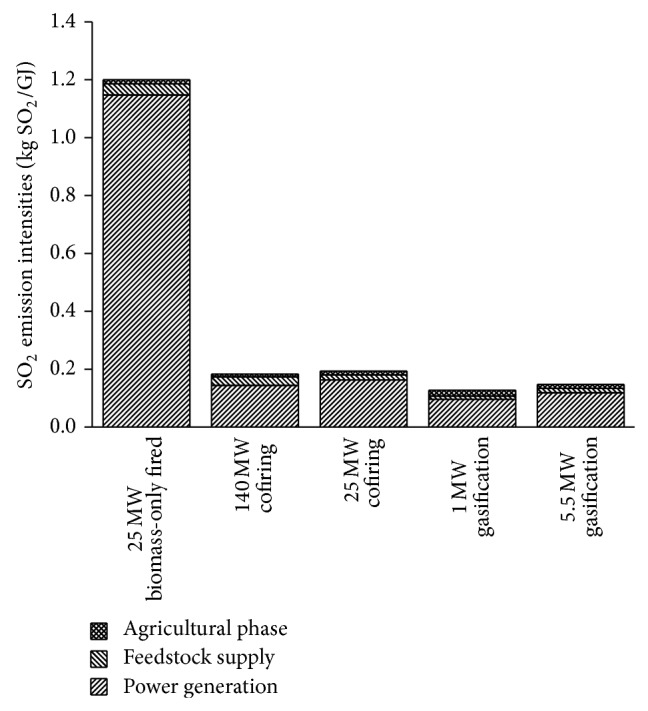
Comparison of SO_2_ emission intensities of BPG systems.

**Figure 5:**
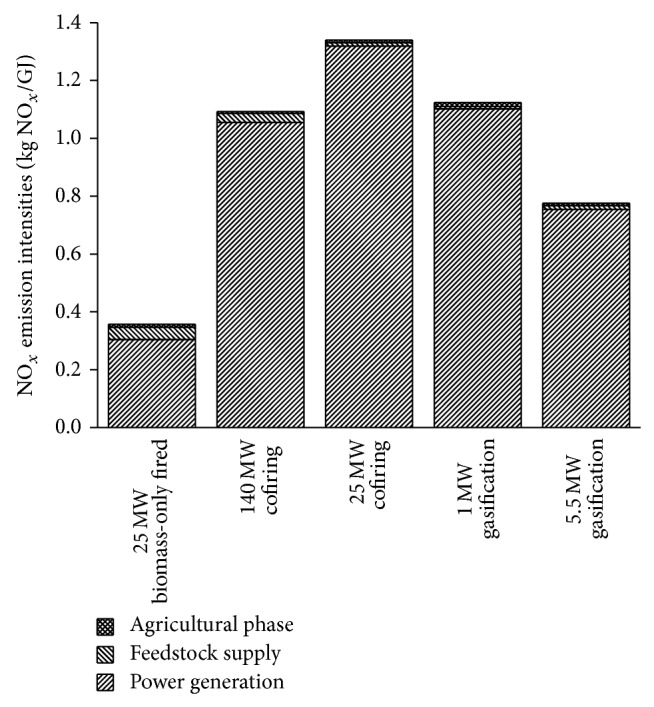
Comparison of NO_*x*_ emission intensities of BPG systems.

The SO_2_ (or NO_*x*_) emission intensity of BPG systems is defined as the SO_2_ (or NO_*x*_) emission by the system per electric energy from biomass delivered to the utility grid. The SO_2_ emission intensity of BPG systems is 0.13–1.20 kg SO_2_/GJ. The SO_2_ emission intensity of BPG systems is 0.36–1.34 kg NO_*x*_/GJ. For all systems, the majority of SO_2_ and NO_*x*_ emission comes from the combustion of biomass fuel, as shown in Figures 4 and 5.

The sulfur content of biomass is much lower than that of coal. On the other hand, desulphurization involves a large amount of investment. For these two reasons, desulphurization device is not usually equipped in the biomass-only fired system in China. Desulphurization device of the coal-fired power plant can effectively remove the SO_2_ for the cofiring system, just as the water scrubber does for the biomass gasification systems. As expected, the worst SO_2_ emission performance is that of the 25 MW biomass-only system.

Comparing the SO_2_ emission intensities of 1.79 kg SO_2_/GJ of the electricity sector obtained by the hybrid LCI model, BPG systems can anyhow provide a significant reduction in SO_2_ emissions, due to the very low sulfur content of biomass. Despite the relatively low SO_2_ emissions intensities, the cofiring systems exhibit a small capacity for reducing SO_2_ emissions (Table 13). An explanation can be given by the assumptions made in this study: the SO_2_ emission factor of the cofiring system was 10%, while only 48% of coal power plants were estimated to use desulphurization in the calculation associated with electricity sector.

NO_*x*_ emission of BPG systems is mainly from biomass fuel combustion. The NO_*x*_ formation is influenced by many factors such as combustion temperature, concentration of oxygen, residence time, and the content of fuel-bound nitrogen. The cofiring systems and the biomass gasification systems can slightly decrease the NO_*x*_ emission, whereas the 25 MW biomass-only fired system performs significantly better in NO_*x*_ emission reductions, despite the fact that no denitrification measures are considered (Table 13).

## 5. Conclusions

Methodological constraints of process-based life-cycle analysis, particularly a problem associated system boundary selection, may lead to some uncertainties in the LCI results. A hybrid LCI framework can ensure the completeness of system boundary and provide a desirable method for quantifying a system's environmental footprint.

There are currently a number of biomass power plants in China. The government has not offered any guidance on preferred type, leaving the market open. A comparative study is necessarily important. In this paper, a hybrid LCI model is used to comparatively evaluate five BPG systems, which may represent the present state-of-the-art in China. A preliminary feasibility estimation of the biomass power in China is provided in terms of primary energy (fossil energy) savings, GHG emission reductions, and avoided emissions of SO_2_ and NO_*x*_.

To get 1 GJ electricity from corn stovers, only 0.11–0.28 GJ of primary energy (PE) is consumed by BPG systems, whereas primary energy as much as 4.42 GJ can be saved by substituting conventional electricity. At the same time, the BPG systems only contribute 26–44 kg CO_2_ eq of GHG emissions, while up to 517 kg CO_2_ eq of GHG missions can be avoided. The cofiring systems, especially the 1 MW cofiring system, can achieve the highest PE savings and GHG emission reductions per electric energy from biomass delivered to the utility grid. Moreover, the PE savings and GHG emission reductions are accomplished at a lower cost of biomass resource and monetary investment. Thus the cofiring systems give better behavior than the biomass-only fired system and the biomass gasification systems. For all systems, the life stage of power generation is responsible for the largest share of PE consumptions and GHG emissions. N_2_O emission from combustion of biomass fuels has made a significant contribution to GHG emission. Another important aspect that should be addressed is the significant contributions of infrastructure, equipment, and maintenance of the plant, which may be easily ignored in a process-based LCI. Inputs of various types of fossil fuels, materials, and services are required in construction and operation of a biomass plant. And the consequent PE consumptions and GHG emissions should be taken into consideration.

The emission intensities of SO_2_ and NO_*x*_ of BPG systems are evaluated to be 0.13–1.20 kg SO_2_/GJ and 0.36–1.34 kg NO_*x*_/GJ, respectively, the majority of which come from the combustion of biomass fuels. Compared with conventional electricity, emission reductions of SO_2_ and NO_*x*_ can be achieved by all BPG systems.

The innovative base of comparison between BPG systems has allowed assessment and comparison of the five electricity production system alternatives with agricultural residues. From the case presented, it is shown that BPG systems could be a high-potential alternative for electricity generation. In addition to the environmental benefits quantified in this LCI, BGP systems will provide other benefits as they are deployed in China, such as rural economic development through the creation of new markets and jobs. A specific LCI study should take into consideration local conditions such as the normal routes of biomass disposal and energy consumption structure, which may have significant effects on results. Moreover, it has to be noted that, by expanding the scope of analysis in hybrid LCA, the level of precision is lost due to the use of highly coarse and aggregated data in input-output table that involved significant amounts of uncertainties and assumptions. The choice of process parameters and allocation procedures can have significant effects on results as well. Although much work is currently being undertaken to determine several values used in this analysis in a more precise way, the main conclusion that can be highlighted is that, based on the values and assumptions used, the cofiring system is more beneficial than biomass-only fired power plant and biomass gasification system, when the PE savings and GHG emission reductions are taken into account.

## Figures and Tables

**Table 1:** Coefficient matrices for the hybrid LCI analysis.

		BPG systems		IO sector		Functional unit	Total output
		Process commodity (*c* = 1,2,…, *l*)	Process activity (*p* = 1,2,…, *m*)	Energy sector (*i* = 1,2,…, *n*_*e*_)	Nonenergy sector (*j* = 1,2,…, *n*)		
BPG systems	Process commodity (*c* = 1,2,…, *l*)		**U** ^*CP*^			**F**	**Q**
	Process activity (*p* = 1,2,…, *m*)	**V** ^*PC*^		**V** ^*PE*^	**V** ^*PN*^		**G**

IO sector	Energy sector (*i* = 1,2,…, *n*_*e*_)		**U** ^*EP*^	**U** ^*EE*^	**U** ^*NE*^		**x**_*E*_
	Nonenergy sector (*j* = 1,2,…, *n*)		**U** ^*NP*^	**U** ^*NE*^	**U** ^*NN*^		**x**_*N*_

**Table 2:** Accounting framework for BPG systems.

		BPG systems		IO sector		Functional unit	Total output
		Process commodity (*c* = 1,2,…, *l*)	Process activity (*p* = 1,2,…, *m*)	Energy sector (*i* = 1,2,…, *n*_*e*_)	Nonenergy sector (*j* = 1,2,…, *n*)		
BPG systems	Process commodity (*c* = 1,2,…, *l*)	**A**_*ff*_	**B** ^*CP*^	**A**_*fn*_	**A**_*fn*_	**F**	**Q**
	Process activity (*p* = 1,2,…, *m*)	**D** ^*PC*^					**G**

IO sector	Energy sector (*i* = 1,2,…, *n*_*e*_)	**A**_*ef*_		**A**_*ee*_	**A**_*en*_		**x**_*E*_
	Nonenergy sector (*j* = 1,2,…, *n*)	**A**_*nf*_		**A**_*ne*_	**A**_*nn*_		**x**_*N*_

**Table 3:** Classifications of IO sectors in hybrid LCI model.

Sector code	Sectors
1-1	Raw coal
1-2	Cleaned coal
1-3	Other washed coal
1-4	Coke
1-5	Coke oven gas
1-6	Other gases
1-7	Other coking products
1-8	Crude oil
1-9	Gasoline
1-10	Kerosene
1-11	Diesel oil
1-12	Fuel oil
1-13	Liquefied petroleum gas
1-14	Refinery gas
1-15	Other petroleum products
1-16	Natural gas
1-17	Electricity
1-18	Heat
2	Farming, forestry, animal husbandry, fishery, and water conservancy (agriculture)
3	Ferrous and nonferrous metals mining and dressing
4	Nonmetal and other minerals mining and dressing
5	Food processing, food production, beverage production, and tobacco processing
6	Textile
7	Garments and other fiber products, leather, furs, and down and related products
8	Timber processing, bamboo, cane, palm and straw products, and furniture manufacturing
9	Papermaking and paper products, printing and record medium reproduction, and cultural, educational, and sports articles
10	Raw chemical materials and chemical products, medical and pharmaceutical products, and chemical fiber, rubber, and plastic products
11	Nonmetal mineral products
12	Smelting and pressing of ferrous and nonferrous metals
13	Metal products
14	Ordinary machinery, equipment for special purpose
15	Transport equipment
16	Electric equipment and machinery
17	Manufacture of communication equipment, computers, and other electronic equipment
18	Instruments, meters, and cultural and office machinery
19	Artwork and other manufacturing
20	Recycling and disposal of waste
21	Water production and supply
22	Construction
23	Transport, storage, postal, and telecommunications services
24	Wholesale, retail trade, hotels, and catering service
25	Other service activities

**Table 4:** Allocation of energy consumptions for energy sectors.

Energy-related sectors in IO table and energy statistical yearbook	Energy sectors in hybrid LCI model	Allocation method of energy consumptions
Coal mining and dressing	1-1 Raw coal 1-2 Cleaned coal 1-3 Other washed coal	Ratio of energy consumptions of raw coal (1-1) and washed coal (1-2, 1-3) was assumed to be 25 : 9, based on Grade 3 of clean production standard of coal mining and processing industry [24]

Petroleum and natural gas extraction	1-8 Crude oil 1-16 Natural gas	Crude oil and refinery gas are consumed in Crude oil extraction. Natural gas is consumed in natural gas extraction

Petroleum processing, coking, and processing of nuclear fuel	1-4 Coke 1-5 Coke oven gas 1-6 Other gases 1-7 Other coking products 1-9 Gasoline 1-10 Kerosene 1-11 Diesel oil 1-12 Fuel oil 1-13 Liquefied petroleum gas 1-14 Refinery gas 1-15 Other petroleum products	Coking products are consumed in coking. Crude oil and refinery gas are consumed in processing of petroleum. Ratios of refining efficiency of gasoline, kerosene, diesel, liquefied petroleum gas, and fuel oil are assumed to be 85% : 87% : 89% : 93.5% : 95% [25]. Refining efficiency of refinery and other petroleum are assumed to be the same as fuel oil

Electric power and steam production and supply	1-17 Electricity 1-18 Steam	The equivalent value of electricity to heat is assumed to be 2.78 [14]

Gas production and supply	1-5 Coke oven gas 1-13 Liquefied petroleum gas 1-16 Natural gas	

**Table 5:** GHG emissions of nonenergy use from industrial processes.

Sector code	Sector category	Industrial processes	GHG emissions
10	Raw chemical materials and chemical products, medical and pharmaceutical products, and chemical fiber, rubber, and plastic products	Manufacturing of ammonia, soda ash, and calcium carbide	105.78 Mt CO_2_

11	Nonmetal mineral products	Manufacturing of cement and plain grass	683.93 Mt CO_2_

12	Smelting and pressing of ferrous and nonferrous metals	Smelting and pressing of ferrochromium, silicon metal and ferro-unclassified, and coke as a reducing agent	873.59 Mt CO_2_

2	Farming, forestry, animal husbandry, fishery, and water conservancy (agriculture)	Enteric fermentation, manure management, rice cultivation, and field burning of agricultural residues	18.44 Mt CH_4_

1-1	Raw coal	Coal mining	19409.97 kt CH_4_

1-8, 1-16	Crude oil, natural gas	Oil and natural gas systems	258.31 kt CH_4_

2	Farming, forestry, animal husbandry, fishery, and water conservancy (agriculture)	Manure management, cropland, and field burning of agricultural residues	614.97 kt N_2_O

10	Raw chemical materials and chemical products, medical and pharmaceutical products, and chemical fiber, rubber, and plastic products	Nitric acid, adipic acid	74.55 kt N_2_O

**Table 6:** NO_*x*_ emissions of nonenergy use from industrial processes.

Category	Industrial processes	Quantity (Mt)	NO_*x*_ emission factors (t/t)	NO_*x*_ emissions (Mt)
10 raw chemical materials and chemical products, medical and pharmaceutical products, and chemical fiber, rubber, and plastic products	Nitric acid	2.009	0.012	0.0241
	Adipic acid	0.215	0.0081	0.0017

12 smelting and pressing of ferrous and nonferrous metals	Iron	494.889	0.000076	0.3761
	Ferrochromium-silicon	0.043	0.0117	0.0005
	Silicon metal	0.81	0.0117	0.0095
	Aluminum	9.358	0.00215	0.0201

13 metal products	Steel rolling	60.927	0.00004	0.2437

**Table 7:** Inputs and allocation in agricultural phrase.

Inputs	Plantation inputs (yuan/mu)	Assigned input of corn stover (yuan/GJ)
Seed	26.92	0.308
Chemical fertilizers^a^	88.43	1.013
Farmyard manure	8.66	0.099
Pesticide	7.96	0.091
Agricultural film	2.62	0.030
Field machinery, irrigation, and animal power	55.64	0.637
Fuels	0.03	0.393
Technical service	0.03	0.143
Tools and materials	2.1	0.061
Maintenance	1.27	0.101
Others	0.12	0.000

Note: ^a^the amount of N-fertilizer applied in physical unit is 10.27 kg N/mu [21]. An emission rate of 1.3% of N-fertilizer for N_2_O [25] was adopted. On the other hand, emissions associated with land use change were not taken into account in this study. The average exchange rate of currency in 2007, 1 yuan = 0.132 USD and 1 yuan = 0.096 EUR.

**Table 8:** Major parameters for biomass feedstock supply.

Items	Value
Straw/grain ratio	0.75
Corn production (kg/mu)	422.4
Lower heating value (LHV) of corn stover (MJ/kg, dry basis)	15.6
Moisture content of corn stover (wt%)	10
Sulfur content of corn stover^a^ (wt%)	0.21
Distribution density of biomass^b^ (t/km)	103.3
Average transport distance from straw-receiving station to the plant^c^ (km)	30
CO_2_ emission factor of diesel^d^ (g/GJ)	74100
SO_2_ emission factor of diesel^e^ (g/GJ)	93.78
NO_*x*_ emission factor of diesel^f^ (g/GJ)	643.19

Note: ^a^an average value of sulfur content of corn stover from Cuiping et al.[26] was adopted to ensure that the BPG systems were comparable.

^b^The transport distance for centralized pattern was calculated using a farmland coverage rate of 0.7 and a availability factor of 0.4 in the model [22]. It was assumed that the collection area is assumed to be a circle centered at the straw-receiving station and the centralized storage site, where the straw is evenly distributed [22].

^c^The average transport distance is used for the distributed pattern in the cases of 25 MW biomass-only fired and 140 MW cofiring.

^d^CO_2_, CH_4_, and N_2_O emission factors for diesel utilization were adopted from IPCC road transport default values, the latter two of which are 3.9 g/GJ [18].

^e^The Chinese specific value for sulfur content of diesel was taken from Song [27]. And the emission rate of sulfur was assumed to be 100%.

^f^The Chinese specific value ofNO_*x*_ emission for diesel vehicles is 27.4 kg/t [28].

**Table 9:** Major parameters for biomass power plant.

Items	25 MW biomass-only fired	140 MW cofiring	25 MW cofiring	1 MW gasification	5.5 MW gasification
Electric efficiency^a^	25.6%	35.4%	27.6%	18.0%	27.0%
Electric efficiency before cofiring		36.1%	28.0%		
Cofiring ratio by energy		20%	15%		
Auxiliary power consumption rate^b^	8%	10%	8%	10%	10%
Annual operating hours (h)	6500	7000	7000	6000	6000
Annual power supply^c^ (GJ)	538200	571536	78246	19440	106920
Life expectancy (year)	15	10	10	15	15
Capital investment (10^4^ yuan)^d^	24145	8413	1155	428	3350
Annual cost (10^4^ yuan)^e^	3255	1148	230	92	572
CH_4 _emission^f^ (kg/GJ biomass)	0.0037	0.0037	0.0037	0.0037	0.0037
N_2_O emission^f^ (kg/GJ biomass)	0.0105	0.0105	0.0105	0.0105	0.0105
SO_2_ emission^g^ (kg/GJ biomass)	0.2393	0.0299	0.0299	0.0008	0.0008
NO_*x*_ emission^h^ (kg/GJ biomass)	0.0590	0.3291	0.3300	0.1733	0.1733

^a^Utility boiler efficiency decrease: 1% for each 10% of coal replaced by biomass (on an energy basis) [2].

^b^Energy consumption of biomass crushing is included.

^c^The energy inputs of the boiler in cofiring plant remain the same as before. The biomass-related power output was listed as the annual power supply of cofiring plant.

^d^Investment on feedstock supply system is not included.

^e^The annual cost consists of depreciation cost, maintenance, materials, and personnel. The cost of feedstock supply is not included.

^f^Since all the carbon in the biomass is recycled, it has been assumed that biomass fuel combustion does not produce GHG emissions due to CO_2_. The CH_4_ and N_2_O emission factors of biomass-only fired plant and biomass gasification system were taken from Wang [25]. In addition, the CH_4_ and N_2_O emission of internal engine is assumed to be the same as that of IGCC from Wang [25]. As in the cofiring case, the emission factors of CH_4_ and N_2_O were assumed to be the same as that of biomass-only fired system.

^g^For biomass gasification power plant, the SO_2_ emission may vary significantly from one to another. The data of the 5.5 MW gasification system adopted here is converted from Jia [29]. And the 1 MW gasification system is assumed to have the same emission. Desulphurization device is not usually commissioned in a biomass-only fired plant, since sulfur content of biomass is usually low. The SO_2_ emission of biomass-only fired plant is estimated to be 80% of that of feedstock. On the other hand, the SO_2_ emission factor of cofiring plant is estimated to be 10%, for desulphurization is commonly used. The sulfur content of coal in the cofiring case is 1.29 wt% [16].

^h^The NO_*x*_ emission factor of biomass-only fired plant was converted from Liu et al. [8]. For biomass gasification power plant, the NO_*x*_ emission is converted from Jia [29]. And the 1 MW gasification system is assumed to have the same NO_*x*_ emission factor. NO_*x*_ emissions reduction can be achieved by cofiring and is calculated using an equation from Tillman [30], which can be expressed as RNO_*x*_ = 0.0008*C* ^2^ + 0.0006*C* + 0.075, where *C* is the percentage biomass cofiring on a calorific basis. The NO_*x*_ emission factor of coal before cofiring is 335.94 g/GJ coal [16].

**Table 10:** Major expenditures and corresponding IO sectors.

Inputs	Sector category
Diesel	1-11 diesel
Electricity	1-17 electricity
Boiler, steam turbine, internal gas engine, handling equipment, air blower, drying equipment, and auxiliary equipment	14 ordinary machinery, equipment for special purpose
Transport vehicles	15 transport equipment
Generator, electricity transmission, and distribution equipment	16 electric equipment and machinery
Construction engineering, wiring, piping, and installation of electric equipment	22 construction
Transportation of equipment and materials^a^	23 transport, storage, postal, and telecommunications services
Technical service, insurance	25 other service activities

^a^The freight and miscellaneous charges of boiler, internal engine, steam turbine, and generator set were evaluated to be 0.6% of the purchase cost. The freight and miscellaneous charges of other equipment and materials were evaluated to be 7%.

**Table 11:** Energy saving performance of BPG systems.

Items	25 MW biomass-only fired	140 MW cofiring	25 MW cofiring	1 MW gasification	5.5 MW gasification
Energy savings^a^ (GJ/GJ)	2.57	3.44	4.42	2.68	2.66
Cost of energy saving (yuan/GJ)	29.5	10.5	8.3	21.8	22.3
Cost of energy saving (GJ biomass/GJ)	1.66	0.99	0.98	2.30	1.55

^a^In the case of cofiring systems, coal transportation in reference system was not taken into consideration.

**Table 12:** GHG emission reduction of BPG systems.

Items	25 MW biomass-only fired	140 MW cofiring	25 MW cofiring	1 MW gasification	5.5 MW gasification
GHG emission reductions (kg CO_2_ eq/GJ)	220	404	517	224	229
Cost of GHG emission reductions (yuan/kg CO_2_ eq)	0.34	0.09	0.07	0.26	0.26
Cost of GHG emission reductions (GJ biomass/kg CO_2_ eq)	0.019	0.008	0.008	0.028	0.018

**Table 13:** SO_2_ and NO_*x*_ emission reductions of BPG systems.

Items	25 MW biomass-only fired	140 MW cofiring	25 MW co-firing	1 MW gasification	5.5 MW gasification
SO_2_ emission reductions (kg SO_2_/GJ)	0.59	0.46	0.62	1.67	1.64
NO_*x*_ emission reductions (kg NO_*x*_/GJ)	0.80	0.21	0.30	0.04	0.39
